# Transcriptome Analysis by Illumina High-Throughout Paired-End Sequencing Reveals the Complexity of Differential Gene Expression during *In Vitro* Plantlet Growth and Flowering in *Amaranthus tricolor* L.

**DOI:** 10.1371/journal.pone.0100919

**Published:** 2014-06-25

**Authors:** Shengcai Liu, Huaqin Kuang, Zhongxiong Lai

**Affiliations:** Institute of Horticultural Biotechnology, Fujian Agriculture and Forestry University, Fuzhou, Fujian, China; The University of Hong Kong, Hong Kong

## Abstract

*Amaranthus tricolor* L. is a C_4_ plant, which is consumed as a major leafy vegetable in some tropical countries. Under conditions of high temperature and short daylight, *Am. tricolor* readily bolts and blooms, degrading leaf quality. A preliminary *in vitro* flowering study demonstrated that the flowering control pathway in *Am. tricolor* may differ from that of *Arabidopsis*. Nevertheless, no transcriptome analysis of the flowering process in *Amaranthus* has been conducted. To study *Am. tricolor* floral regulatory mechanisms, we conducted a large-scale transcriptome analysis—based on Illumina HiSeq sequencing of cDNA libraries generated from *Am. tricolor* at young seedling (YSS), adult seedling (ASS), flower bud (FBS), and flowering (FS) stages. A total of 99,312 unigenes were obtained. Using BLASTX, 43,088 unigenes (43.39%) were found to have significant similarity with accessions in Nr, Nt, and Swiss-Prot databases. Of these unigenes, 11,291 were mapped to 266 KEGG pathways. Further analysis of the four digital transcriptomes revealed that 735, 17,184, 274, and 206 unigenes were specifically expressed during YSS, ASS, FBS, and FS, respectively, with 59,517 unigenes expressed throughout the four stages. These unigenes were involved in many metabolic pathways related to *in vitro* flowering. Among these pathways, 259 unigenes were associated with ubiquitin-mediated proteolysis, indicating its importance for *in vitro* flowering in *Am. tricolor*. Other pathways, such as circadian rhythm and cell cycle, also had important roles. Finally, 26 unigenes were validated by qRT-PCR in samples from *Am. tricolor* at YSS, ASS, FBS, and FS; their differential expressions at the various stages indicate their possible roles in *Am. tricolor* growth and development, but the results were somewhat similar to *Arabidopsis*. Because unigenes involved in many metabolic pathways or of unknown function were revealed to regulate *in vitro* plantlet growth and flowering in *Am. tricolor*, the process appears to be highly complex in this species.

## Introduction

*Amaranthus* L., a genus of approximately 70 C_4_ dicotyledonous herbaceous plant species, is widely distributed throughout warm temperate and tropical regions of the world [1], [2]. Many amaranth species are economically important crop plants valued for their nutritional and horticultural significance [3]. The “grain amaranths” are a major source of highly nutritious pseudocereals that complement cereal diets [3], [4], while “green amaranths” are savored as a major leafy vegetable in several Asian and African countries, such as India, Bangladesh, and Zimbabwe [3], [5]–[8]. Some species are also valued for their ornamental, forage, or pharmaceutical properties. Leafy amaranths, with axillary determinate inflorescences and native to southeast Asia, constitute one of the most widely consumed tropical vegetable crops. Most of these *Amaranthus* species, especially *Am. tricolor*, are cultivated as green vegetables similar to spinach, broccoli, and cabbage [3], contributing to a wholesome diet. The leaves, along with the stems, may be eaten as a salad vegetable. In Africa, amaranth is usually cooked as a leafy vegetable. The mucilaginous leaves are rich in vitamins and minerals. They also contain betalain pigments, used for food coloring, that possess antioxidant, anticancer, antiviral, antiparasitic, and radical scavenging properties against certain oxidative stress-related disorders [9]–[12].

*Am. tricolor* readily bolts and blooms under high-temperature, short daylight conditions, which leads to deterioration in quality and prevents year-round production. To preserve fresh quality and increase production, growth can be regulated to maintain vegetative growth and delay blooming. Investigation of *in vitro* flowering in *Am. tricolor* is very useful for horticultural production, and more importantly, *in vitro* plantlets of *Am. tricolor* are ideal experimental materials for the study of *in vitro* flowering mechanisms [13].

Flowering is an important transformative event in the life cycle of higher plants. The process is central to individual plant development, as proper floral initiation timing is essential for optimal production of fruits and seeds to ensure reproductive success. Studies of plant flowering regulatory mechanisms have primarily focused on *Arabidopsis thaliana*. These studies have revealed the complexity of the flowering process, which is controlled by genetic and environmental factors [14], [15]. The expression and interaction of multiple genes is required to accurately regulate flowering times and, ultimately, floral development [16], [17]. Nevertheless, floral induction mechanisms uncovered in *Arabidopsis* are not adequate to fully explain floral regulation in short-day plants [16], [18]. Investigation of flowering mechanisms in the short-day plant *Am. tricolor* can aid further elucidation of the flowering process.

Transcriptome analysis is a general phenotyping method [19], [20]. Using this technique, a large number of functional genes can be identified, and genes differently expressed among samples of the same species can be uncovered. Transcriptome analysis is particularly useful for revealing relationships between plant gene expression and phenotypes. Application of transcriptome sequencing technology to study plant developmental and floral transition processes has generated large quantities of flowering-related data [21]–[29]. The results of such studies have demonstrated that this method is effective for investigating the transformations of plant growth and development.

The number of advanced genomic or transcriptomic studies of the genus *Amaranthus* has recently increased, with at least seven published reports appearing since 2008. One such report detailed the construction of a bacterial artificial chromosome (BAC) library for *Am. hypochondriacus* [30]. Using this BAC library, microsatellite markers for *Amaranthus* were subsequently developed and applied to clarify taxonomic relationships within the *Am. hybridus* complex [31], [32].

*De novo* whole genome sequencing is an effective approach for genomic study of non-model species with unsequenced genomes [33]. Using next-generation 454 pyrosequencing technology, for example, the whole genome of *Am*. *tuberculatus* has been sequenced and used to identify herbicide-resistant genes and develop molecular markers for population genetics and phylogenetics studies [34]. The transcriptome of this species has also been sequenced, with 44,469 unigenes uncovered [35]. Thousands of single nucleotide polymorphisms (SNPs) in four different populations of *Am. caudatus* have been identified via genomic reduction, barcoding, and 454-pyrosequencing [36]. Next-generation 454 pyrosequencing technology has also been used to generate leaf and stem gene expression profiles for *Am. hypochondriacus* and to analyze responses to biotic and abiotic stress [2]. In that study, the generated *Am. hypochondriacus* transcriptome was also compared with that of *Am. tuberculatus* to highlight differences in drought stress response between cereal and weedy *Amaranthus* species.

Although various transcriptome studies, as described above, have been conducted on *Amaranthus* species, the use of this method to explore the flowering process in *Am. tricolor* has not yet been reported. The transcriptome of *Am. tricolor* is worthy of study, as a preliminary analysis of a small RNA library from *Am. tricolor* has suggested that flowering control pathways may differ between *Am. tricolor* and *Arabidopsis* [37]. Examination of the *in vitro* flowering process of *Am. tricolor* would help to lay a foundation for further study of flowering control mechanisms and provide evidence for regulation of growth and development of other *Amaranthus* species, including *Am. hypochondriacus*. The results would also have important significance for the breeding of *Amaranthus* species to enhance the quality of edible organs,both leaves and seeds.

## Results

### Sequencing and *de novo* assembly of the *Am. tricolor* transcriptome during *in vitro* plantlet growth and flowering

To comprehensively cover the *Amaranthus in vitro* flowering transcriptome, total RNA was extracted from four *Am. tricolor* developmental stages: young seedling (YSS), adult seedling (ASS), flower bud (FBS), and flowering (FS) stages. Equal amounts of total RNA from each sample were pooled together. The mRNA was isolated, enriched, sheared into smaller fragments, and reverse-transcribed into cDNA, which was subjected to sequencing on an Illumina HiSeq 2000 sequencing platform. After removal of adaptor sequences, ambiguous reads, and low-quality reads, approximately 319,199,714 high-quality clean reads, 100-bp long and comprising 31,919,971,400 nucleotides (31.92 Gb), were obtained. The raw data were deposited in the NCBI Sequence Read Archive (SRA) under accession numbers SRR924089, SRR924090, SRR924091, and SRR924092. A statistical summary of the data generated by the sequencing process is outlined in Table 1. The number of nucleotides, Q20 percentages (sequencing error rate <1%), and GC percentages obtained from the four developmental stages ranged from 6,203,563,600–13,136,742,400 bp, 94.29–96.73%, and 43.37–47.55%, respectively. These values indicate that the sequencing data was of sufficient quantity and quality to ensure sequence assembly accuracy and adequate transcriptome coverage.

**Table 1:** Statistical summary of reads generated by transcriptome sequencing of *in vitro* plantlets of *Am. tricolor*.

Sample^1^	Read length (bp)	Clean reads	Clean bases (bp)	Q20 (%)	GC (%)
**YSS**	100	62,035,636	6,203,563,600	95.88	44.11
**ASS**	100	131,367,424	13,136,742,400	94.29	47.55
**FBS**	100	62,025,474	6,202,547,400	96.85	43.37
**FS**	100	63,771,180	6,377,118,000	96.73	43.46

1 YSS  =  young seedling stage; ASS  =  adult seedling stage; FBS  =  flower bud stage; FS  =  flowering stage.

All high-quality reads were assembled *de novo* using the Trinity program [38]. Assembly of reads generated 1,161,029–3,172,183 contigs from the four stages of *in vitro* plantlets, with a maximum length of 15,543–18,338 bp, mean length of 63.9–92.41 bp, GC percentage of 41.24–44.32%, and an N50 of 49–148 bp (Table 2). Using the paired-end sequence data, 54,878–110,506 scaffolds were generated, with a maximum length of 13,702–23,783 bp, mean length of 1151.88–1295.08 bp, GC percentage of 38.91–39.70%, and an N50 of 1870–2147 bp (Table 3). These results demonstrate that the sequencing data was sufficiently ample for assembly of longer scaffolds into unigenes.

**Table 2:** Contigs from assembly of reads generated by transcriptome sequencing of *in vitro* plantlets of *Am. tricolor*.

Sample	Number of contigs	Total number of bases (bp)	Maximum length (bp)	Minimum length (bp)	Mean length (bp)	GC%	N50 (bp)
**YSS**	1,472,193	110,479,825	15,543	25	75.04	42.76	60
**ASS**	3,172,183	202,726,925	18,338	25	63.9	44.32	49
**FBS**	1,349,717	116,585,340	15,675	25	86.37	41.30	114
**FS**	1,161,029	107,293,565	17,428	25	92.41	41.24	148

**Table 3:** Scaffolds from assembly of reads generated by transcriptome sequencing of *in vitro* plantlets of *Am. tricolor*.

Sample	Number of scaffolds	Total number of bases (bp)	Maximum length (bp)	Minimum length (bp)	Mean length (bp)	GC%	N50 (bp)
**YSS**	54,878	64,566,359	15,545	201	1176.54	39.70	1870
**ASS**	110,506	127,290,270	23,783	201	1151.88	39.69	2005
**FBS**	90,132	116,728,488	13,702	201	1295.08	38.91	2147
**FS**	88,379	112,398,889	15,471	201	1271.78	39.05	2125

**Figure 1:**
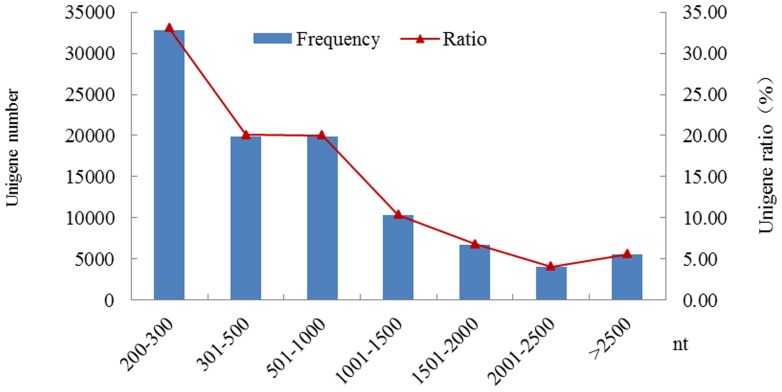
Length distribution of unigenes identified from *Amaranthus tricolor* transcriptomes.

Unigene length is a very important factor that reflects data assembly quality. After further gap filling and exclusion of unigenes less than 200-bp long, the scaffolds from the four plantlet developmental stages were assembled into 99,312 unigenes with a maximum length of 23,783 bp, a mean length of 831.65 bp, a GC percentage of 39.90%, and an N50 of 1,460 bp excluding unknown bases (“N”) (Table 4). Unigenes with lengths ranging from 201–300 bp, 301–500 bp, 501–1,000 bp, 1,001–1,500 bp, 1,501–2,000 bp, 2,001–2,500 bp, and >2,500 bp accounted for 33.05% (32,827), 20.07% (19,931), 20.02% (19,881), 10.37% (10,302), 6.81% (6,760), 4.08% (4,050), and 5,561 (5.60%) of total unigenes, respectively (Figure 1 and Table 5).

**Table 4:** Summary of unigenes generated by transcriptome sequencing of *in vitro* plantlets of *Am. tricolor*.

Item	Number
**Total number of unigenes**	99,312
**Total number of bases (bp)**	82,593,177
**Average unigene length (bp)**	831.65
**Longest unigene length (bp)**	23,783
**Shortest unigene length (bp)**	201
**Average GC percentage (%)**	39.90%
**Non-ACGT bases (bp)**	0
**N50 (bp)**	1,460

**Table 5:** Length distribution of unigenes generated by transcriptome sequencing of *in vitro* plantlets of *Am. tricolor*.

Length Range (bp)	Frequency	Ratio (%)
**201–300**	32,827	33.05
**301–500**	19,931	20.07
**501–1,000**	19,881	20.02
**1,001–1,500**	10,302	10.37
**1,501–2,000**	6,760	6.81
**2,001–2,500**	4,050	4.08
**>2,500**	5,561	5.60

### Functional annotation of genes expressed during *Am. tricolor in vitro* plantlet growth and flowering

For further analysis, open reading frames (ORFs) of unigene sequences with lengths greater than 200 bp were identified using getorf software (http://emboss.bioinformatics.nl/cgi-bin/emboss/getorf). For protein prediction and gene annotation, all assembled unigene sequences were first searched against plant proteins found in the NCBI non-redundant database (Nr), and then against Swiss-Prot, Kyoto Encyclopedia of Genes and Genomes pathway (KEGG), and Clusters of Orthologous Groups of proteins (COG) [39] databases using the BLASTX program (*E*-value <1×10^−5^). A total of 43,088 significant BLAST hits (43.39% of all unigenes) were returned. Among them, 42,592 unigenes (52.89% of all unigenes) had hits in the Nr database, and 27,496 unigenes (27.69% of all unigenes) were annotated against the Swiss-Prot database. These results reveal that many genes of unknown function play important regulatory roles during *in vitro* flowering in *Am. tricolor*, and that this process is unexpectedly complex. Unigenes with lengths ranging from 201–500 bp, 501–1,000 bp, 1,001–1,500 bp, 1,501–2,000 bp, 2,001–3,000 bp, and >3,000 bp accounted for 22.54% (9,710), 23.13% (9,968), 19.08% (8,221), 14.20% (6,120), 13.71% (5,907), and 7.34% (3,162), respectively, of the 43,088 annotated unigenes.

### Gene Ontology functional analysis of genes expressed during *Am. tricolor in vitro* plantlet growth and flowering

**Figure 2:**
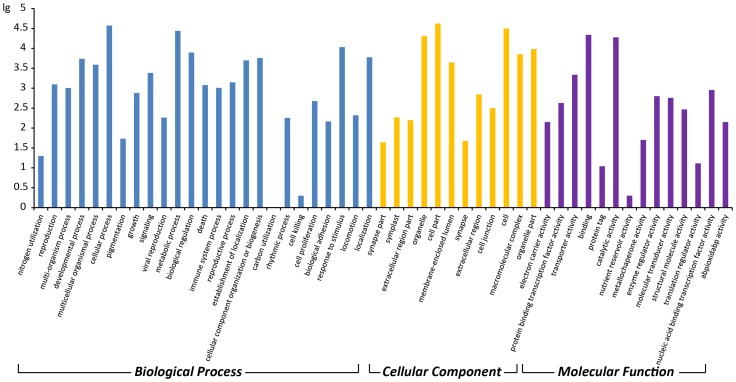
Gene Ontology (GO) functional classification of genes expressed during *Am. tricolor in vitro* plantlet growth and flowering.

Gene Ontology (GO) is an international standardized gene-function classification system that uses a dynamically updated, controlled vocabulary and a strictly defined concept to comprehensively describe the properties of genes and their products in any organism [40]. GO terms, which are assigned to unigenes based on sequence similarities to known proteins in the UniProt database annotated with GO terms as well as their InterPro and Pfam domains, are the basic GO units. The GO database comprises three ontologies: molecular function, cellular components, and biological processes. A total of 282,582 unigenes were assigned into the three main GO categories and 51 sub-categories (functional groups). A high percentage of genes in the biological processes category fell under “cellular process” (37,279), “metabolic process” (27.575), and “response to stimulus” (10,796). Genes related to “cell part” (42,055), “cell” (31,611), and “organelles” (20,336) were heavily represented in the cellular components category, while “binding” and “catalytic activity” subcategories dominated the molecular function category (Figure 2 and Table 6).

**Table 6:** Gene Ontology functional classification of unigenes from *in vitro* plantlets of *Am. tricolor*.

Biological Process					
**Description**	Total	YSS	ASS	FBS	FS
**Nitrogen utilization**	20	16	18	17	17
**Reproduction**	1,249	1,007	1,222	1,001	1,002
**Multi-organism process**	1,015	841	998	827	832
**Developmental process**	5,454	4,474	5,383	4,459	4,465
**Multicellular organismal process**	3,867	3,143	3,816	3,130	3,136
**Cellular process**	37,279	25,677	36,824	25,606	25,671
**Pigmentation**	54	37	52	35	37
**Growth**	756	578	743	574	570
**Signaling**	2,415	1,985	2,385	1,960	1,969
**Viral reproduction**	183	126	181	124	124
**Metabolic process**	27,575	18,547	27,258	18,505	18,566
**Biological regulation**	7,860	6,215	7,779	6,205	6,213
**Death**	1,196	1,021	1,189	1,014	1,014
**Immune system process**	1,022	833	1,013	821	821
**Reproductive process**	1,400	1,170	1,367	1,163	1,169
**Establishment of localization**	4,984	3,291	4,954	3,270	3,273
**Cellular component organization or biogenesis**	5,721	3,352	5,642	3,342	3,332
**Carbon utilization**	1	0	1	0	0
**Rhythmic process**	180	168	175	152	165
**Cell killing**	2	2	2	2	2
**Cell proliferation**	474	368	469	364	366
**Biological adhesion**	147	102	147	102	102
**Response to stimulus**	10,796	8,617	10,631	8,568	8,596
**Locomotion**	209	163	209	161	162
**Localization**	5,941	3,775	5,893	3,738	3,739

Cellular Component					
**Description**	Total	YSS	ASS	FBS	FS
**Synapse part**	44	33	44	33	33
**Symplast**	184	163	183	161	162
**Extracellular region part**	157	132	157	132	132
**Organelle**	20,336	13,841	20,115	13,743	13,773
**Cell part**	42,055	28,733	41,612	28,502	28,556
**Membrane-enclosed lumen**	4,436	2,961	4,387	2,956	2,962
**Synapse**	47	44	47	44	44
**Extracellular region**	697	554	691	552	555
**Cell junction**	314	264	313	261	262
**Cell**	31,611	21,795	31,268	21,657	21,702
**Macromolecular complex**	7,104	4,714	7,033	4,709	4,715
**Organelle part**	9,590	6,540	9,486	6,528	6,538

Molecular Function					
**Description**	Total	YSS	ASS	FBS	FS
**Electron carrier activity**	142	74	138	73	72
**Protein binding transcription factor activity**	426	269	421	264	267
**Transporter activity**	2,171	1,529	2,147	1,512	1,514
**Binding**	21,874	15,739	21,630	15,696	15,732
**Protein tag**	11	7	11	7	7
**Catalytic activity**	18,979	13,123	18,662	13,070	13,112
**Nutrient reservoir activity**	2	2	2	1	2
**Metallochaperone activity**	50	41	50	41	41
**Enzyme regulator activity**	629	475	618	468	467
**Molecular transducer activity**	572	443	566	431	441
**Structural molecule activity**	292	192	292	190	190
**Translation regulator activity**	13	8	13	8	8
**Nucleic acid binding transcription factor activity**	905	769	887	769	764
**Abpioxidabp activity**	141	106	141	104	106

**Figure 3:**
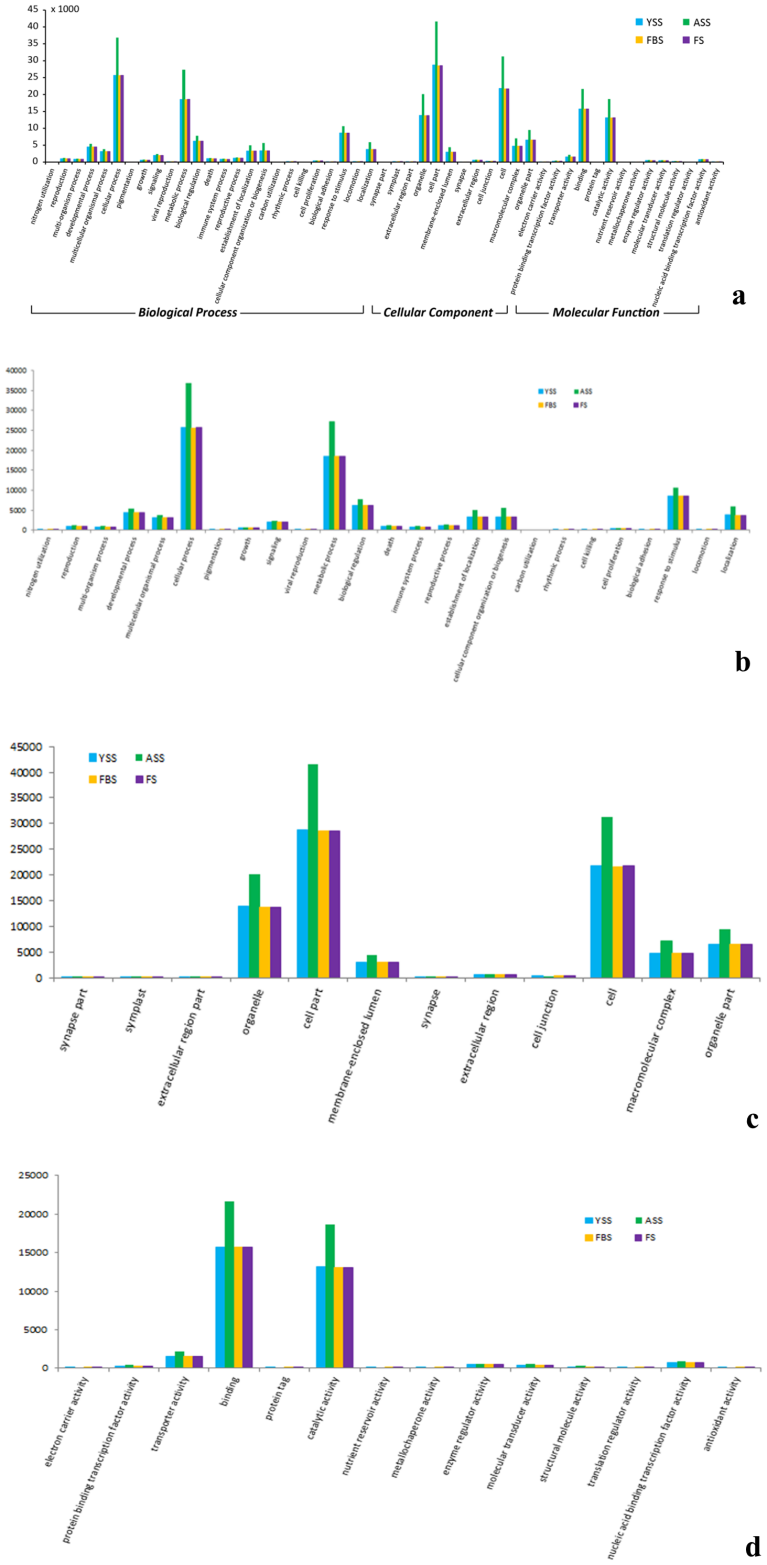
GO functional classification. (a) GO functional classification of genes expressed during each developmental stage in *Am. tricolor*. (b–d) Distribution of expressed genes in each developmental stage associated with the categories of (b) Biological Processes, (c) Cellular Components, and (d) Molecular Function. Developmental stages YSS, ASS, FBS, and FS correspond respectively to young seedling, adult seedling, flower bud, and flowering stages.

Within each subcategory, the number of genes functionally annotated from each developmental stage was similar to the total number of genes annotated (Figure 3a–d and Table 6). These results demonstrate that identified genes associated with molecular function, cellular component, and biological process categories play important regulatory roles during *in vitro* flowering in *Am. tricolor*.

### COG functional classification of genes expressed during *Am. tricolor in vitro* plantlet growth and flowering

**Figure 4:**
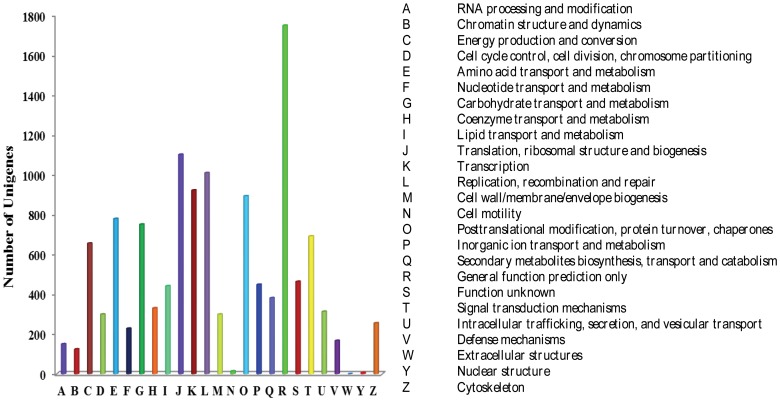
COG functional classification of genes expressed during *Am. tricolor in vitro* plantlet growth and flowering.

The COG database contains classifications of orthologous gene products. Each protein in the COG database is assumed to have evolved from an ancestor protein. The database includes coding proteins of complete genomes as well as information about the evolutionary relationships of bacteria, algae, and eukaryotes. Out of 10,087 unigenes matching the public databases, 12,460 sequences (12.55% of all unigenes) were classified into 24 COG categories (Figure 4 and Table 7). “General function prediction only” represented the largest group (1747; 14.02%), followed by “Translation, ribosomal structure and biogenesis” (1,100, 8.83%), and “Replication, recombination and repair” (1,008, 8.09%). “Cell motility” (14; 0.11%) and “Nuclear structure” (6; 0.05%) were the smallest groups.

**Table 7:** COG functional classification of *Am. tricolor* unigenes.

Code	Number	Function class
**CELLULAR PROCESSES AND SIGNALING**		
**D**	299	Cell cycle control, cell division, chromosome partitioning
**M**	299	Cell wall/membrane/envelope biogenesis
**N**	14	Cell motility
**O**	892	Posttranslational modification, protein turnover, chaperones
**T**	691	Signal transduction mechanisms
**U**	313	Intracellular trafficking, secretion, and vesicular transport
**V**	167	Defense mechanisms
**W**	0	Extracellular structures
**Y**	6	Nuclear structure
**Z**	255	Cytoskeleton
**INFORMATION STORAGE AND PROCESSING**		
**A**	150	RNA processing and modification
**B**	124	Chromatin structure and dynamics
**J**	1,100	Translation, ribosomal structure and biogenesis
**K**	921	Transcription
**L**	1,008	Replication, recombination and repair
**METABOLISM**		
**C**	655	Energy production and conversion
**E**	778	Amino acid transport and metabolism
**F**	228	Nucleotide transport and metabolism
**G**	750	Carbohydrate transport and metabolism
**H**	330	Coenzyme transport and metabolism
**I**	441	Lipid transport and metabolism
**P**	448	Inorganic ion transport and metabolism
**Q**	381	Secondary metabolites biosynthesis, transport and catabolism
**POORLY CHARACTERIZED**		
**R**	1747	General function prediction only
**S**	463	Function unknown

### KEGG functional classification during *Am. tricolor in vitro* plantlet growth and flowering

Pathway-based analysis can help us further understand the biological functions of genes. The KEGG pathway database contains information on networks of intracellular molecular interactions and their organism-specific variations. Once unigenes are assigned KEGG Orthology (KO) identifiers, or K numbers, organism-specific pathway maps and BRITE functional hierarchies are automatically generated. In total, 5,998 unigenes were assigned K numbers, pathway maps, and BRITE functional hierarchies. Among them, 11,291 unigenes were assigned to 266 KEGG pathways, including ubiquitin-mediated proteolysis, circadian rhythm, cell cycle, cytokine-cytokine receptor interaction, oxidative phosphorylation, starch and sucrose metabolism, phototransduction, and ubiquinone and other terpenoid-quinone biosynthesis, and 4,558 unigenes were assigned to 32 KEGG BRITE functional hierarchies, such as ubiquitin system, chromosome, and protein kinases (File S1).

**Figure 5:**
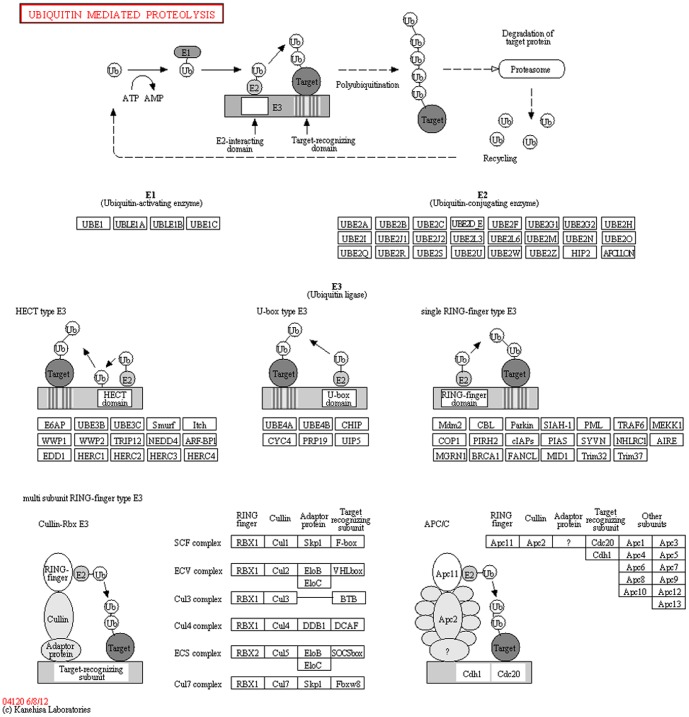
Ubiquitin-mediated proteolysis and associated genes. (from http://www.kegg.jp/kegg-bin/highlight_pathway?scale=1.0&map=map04120&keyword=04120).

**Figure 6:**
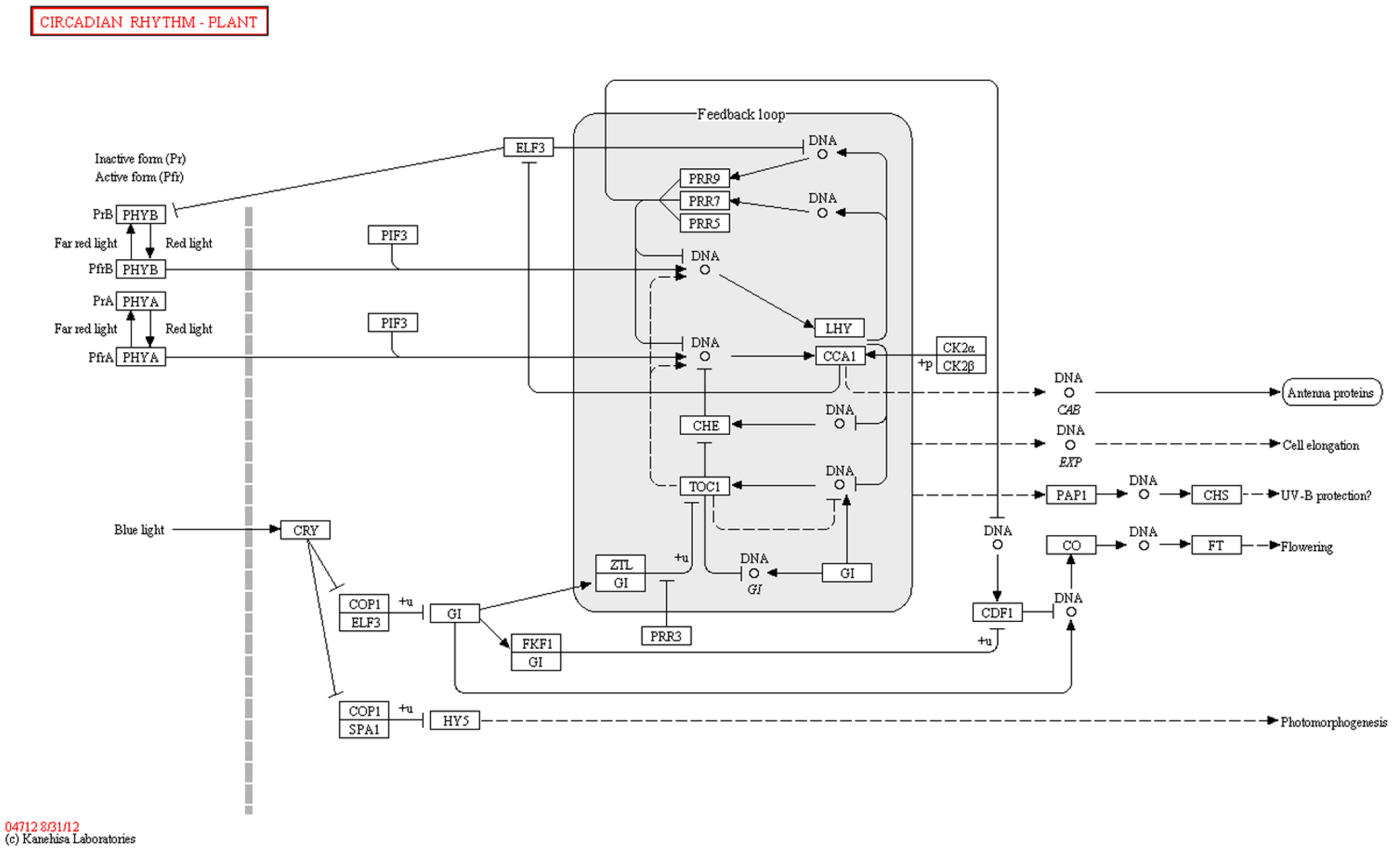
Plant circadian-rhythm pathways and genes. (from http://www.kegg.jp/kegg-bin/highlight_pathway?scale=1.0&map=map04712&keyword=04712).

**Figure 7:**
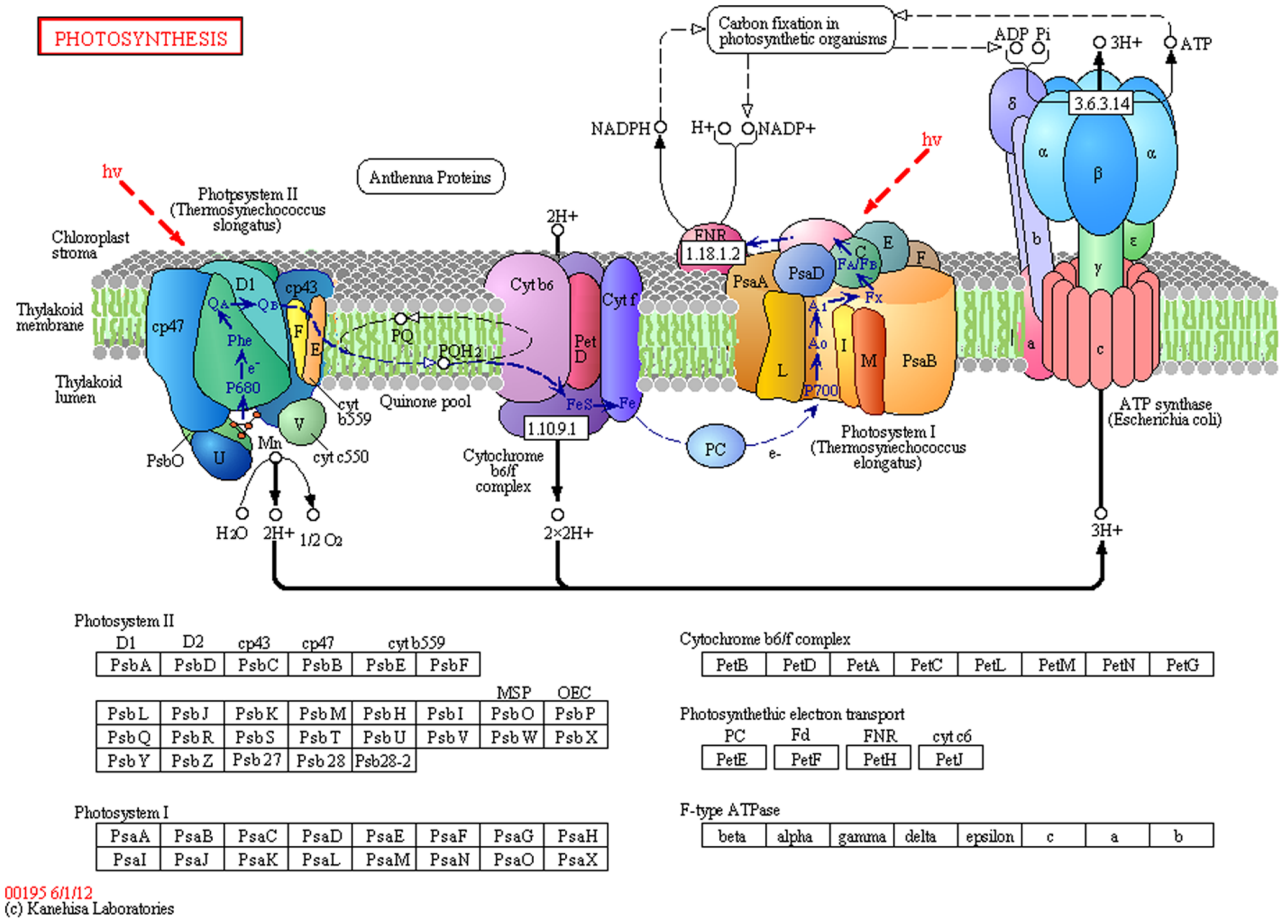
Plant photosynthesis and associated genes. (from http://www.kegg.jp/kegg-bin/highlight_pathway?scale=1.0&map=map00195&keyword=00195).

The ubiquitin-mediated proteolysis pathway (PATH:ko04120; Figure 5) was associated with 259 unigenes, including *UBC1, UBC2, UBC12, COP1, DDB2, TOM1, CHIP, HERC1, CUL1, CPC11*, and *CDC20*. The pathway category of plant circadian rhythm (PATH:ko04712; Figure 6) comprised 196 unigenes, such as *XTH9*, *XTH33*, *ELF3*, *LHY*, *PHYA*, *PHYB*, *PIF3*, and *CRY1*, and the photosynthesis-related pathway category (PATH:ko00195; Figure 7) comprised 41 unigenes, including *ARF*, *PSAN*, *GLP1*, *CRD1*, and *CKI1*. The pentose phosphate pathway (PATH:ko00300) included 32 unigenes, such as *GAPN*, *AIM41*, *RPE*, *PGM*, *SOL1*, and *KPR4*. Phototransduction (ko04744) was associated with 16 unigenes, such as *FRS3*, *CALM*, and *ARR3*. Finally, plant-pathogen interaction pathways (ko04626) were represented by 133 unigenes, including *CERK1*, *RIN4*, *APC1*, *CDPK*, *AGD11*, *SERK4*, *MYB*, and *EFR*.

### Analysis of genes differentially expressed during *Am. tricolor in vitro* plantlet growth and flowering

Unigene expression levels were normalized to the number of reads per kilobase of exon region per million mapped reads (RPKM) [20], [41]. RPKM values of unigenes expressed during YSS, ASS, FBS, and FS varied widely, i.e., 0.00862021–106615, 0.0140538–44980.8, 0.0075633–49467.9, and 0.0141148–59971, respectively (Table 8). These results demonstrate that even extremely small expression level differences can be detected using the Illumina HiSeq 2000 system [42].

**Table 8:** Differentially expressed genes among four developmental stages of *in vitro* plantlets of *Am. tricolor*.

RPKM	YSS (67,449)	ASS (93,842)	FBS (74,866)	FS (73,727)
**<1**	13,124	9,197	8,479	7,537
**1–5**	22,310	39,038	23,768	22,406
**5–10**	11,471	16,934	13,617	13,608
**10–100**	18,405	26,752	26,365	27,400
**100–1,000**	1,953	1,769	2,477	2,616
**>1000**	186	152	160	160

**Figure 8:**
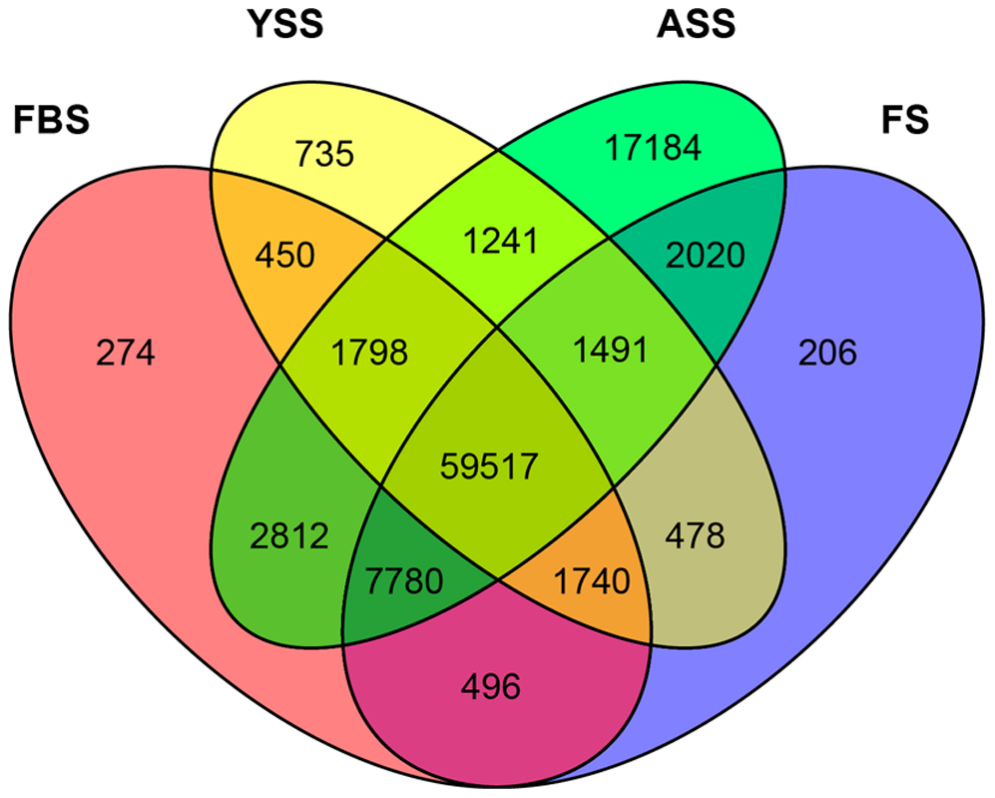
Venn diagram showing shared and unique unigenes from four developmental stages of *Am. tricolor in vitro* plantlet growth and flowering.

Differential gene expression analysis between unigenes from FBS and YSS, FBS and ASS, and FBS and FS was performed using DESeq software [43]. A *p*-value cut-off of <0.05 was used after adjustment for multiple testing using the Benjamini and Hochberg false discovery rate method. A total of 59,517 genes were differentially expressed among the four stages, with 735, 17,184, 274, and 206 genes specifically expressed in YSS, ASS, FBS, and FS, respectively (Figure 8).

We identified 304 genes differentially expressed between FBS and YSS, including 277 and 27 genes that were respectively up- and down-regulatedin FBS. These genes included *YHE5*, *YAI5*, *XTH26*, *UFOG*, *SRG1*, *AP1*, *AGL8*, and *LEC*. In addition, 245 genes were differentially expressed between FBS and ASS, including 187 and 58 genes respectively up- and down-regulated in FBS, such as *XTH9*, *LHY*, *PLCD4*, *HSP70*, *SRG1*, and *CKX3*. Finally, 30 genes were differentially expressed between FBS and FS, including 18 and 12 genes that were up- and down-regulated, respectively, in FBS. These differentially expressed genes included *AG*, *WAX2*, *UFOG*, *PUB23*, *PER57*, *NLTP*, *ITH5*, *ICI1*, *HARB1*, *FAO1*, *ERF17*, and *ACCR4*. Based on this analysis, these genes are inferred to be specifically involved in the process of *in vitro* flowering in *Am. tricolor*, and may have important regulatory roles (Table 9).

**Table 9:** Differentially expressed genes during flowering of *in vitro* plantlets of *Am. tricolor*.

	FBS vs. YSS	FBS vs. ASS	FBS vs. FS
**Total number of unigenes**	78,811	96,800	65,535
**Significantly differentially expressed**	304	245	30
**Not significantly differentially expressed**	78,507	96,555	65,505
**Up-regulated in FBS**	277	187	6
**Down-regulated in FBS**	27	58	12

### Real-time quantitative PCR (qRT-PCR) validation of genes expressed during *Am. tricolor in vitro* plantlet growth and flowering

To confirm that the unigenes obtained from sequencing and computational analysis were indeed expressed and to analyze gene expression profile differences during *in vitro* plantlet growth and flowering in *Am. tricolor*, we used qRT-PCR to detect the expression of 26 unigenes: 10 plant circadian rhythm pathway (PATH:ko04712)-related genes (*XTH9, LHY, ELF3, PHYA, PIF3, TOC1, CCA1, APRR5, APRR1*, and *TIF*), 5 ubiquitin-mediated proteolysis pathway (PATH:ko04120)-related genes (*UBC2, UBC12, TOM1, COP1*, and *CUL1*), and 11 genes associated with other pathways or with the pathway not known (*ARF, PSAN, GLP1, CRD1,AGL8, AP1, MTP, CKX4, EF-1a, CYP*, and *HSP70*).

The photoperiod-dependent flowering pathway is the main flowering pathway, with interactions between light and an internal timekeeping mechanism—the circadian clock—regulating photoperiodic flowering [44], [45]. In the model plant *Arabidopsis*, circadian clock-related genes have been found to regulate the floral transition. Constitutive overexpression of CCA1 delays flowering under short-day conditions [46], [47], whereas *elf3-1*, *cca1*, and *lhy* mutations accelerate such flowering [44], [48]. In addition, CCA1 can interact with other circadian clock-related genes, such as *ELF3* and *LHY*, to regulate the transition to flowering [49], [50]. In our study, expression of the circadian clock-related genes assayed by qRT-PCR (excluding *XTH9*) declined from YSS to FBS, implying that this lowering of gene expression may promote the transition from vegetative to reproductive stages.

**Figure 9:**
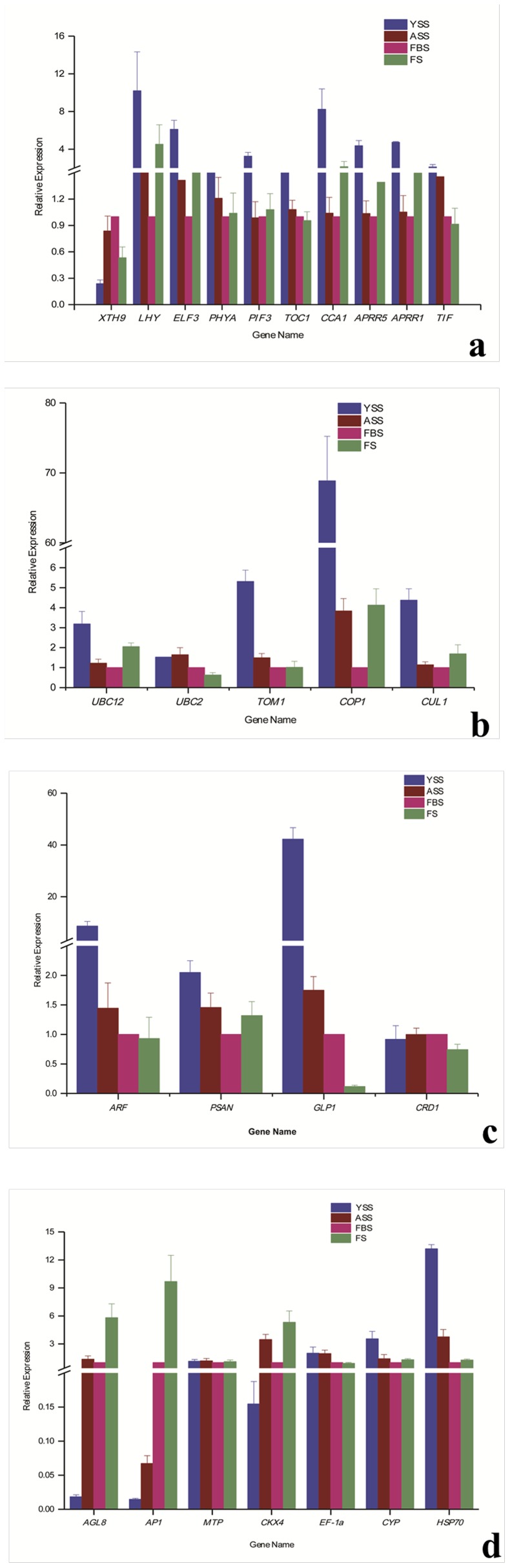
Results of qRT-PCR analysis of 26 candidate unigenes during *Am. tricolor in vitro* plantlet growth and flowering. (a) Plant circadian rhythm pathway-related unigenes. (b) Ubiquitin-mediated proteolysis pathway-related unigenes. (c–d) Genes related to other or unknown pathways.

*XTH9* is expressed in *Arabidopsis* at different development stages, including during differentiation of vegetative and reproductive meristems and cell elongation of inflorescence stems [51]. This gene functions in tissues closest to shoot apices, where cell division is most active. As shown in Fig. 9a, expression of *XTH9* increased from YSS to FBS and then declined, but still remained higher than at YSS. The *XTH9* transcript was detectable during both vegetative and reproductive phases, suggesting a correlation between *XTH9* expression and *Am. tricolor* growth and development.

Numerous studies have suggested that ubiquitin-mediated proteolysis plays an important role in photoperiod-responsive flowering-time regulation. In particular, *COP1* can regulate the entire growth and development process. In rice (*Oryza sativa*), the *COP1* ortholog *PPS* has been confirmed to promote the change from juvenile to adult phases and to also suppress the transition from vegetative to reproductive stages [52]. The *pps-1* mutant is able to both prolong the juvenile phase and to promote early flowering. In our research, relative gene expression of the *COP1* gene declined from YSS to FBS and then increased (Fig. 9b). This result indicates that elevated *COP1* gene expression at YSS promotes the change from YSS to ASS, while lowered expression at FBS promotes the transition from vegetative to reproductive stages. *COP1* may interact with other ubiquitin-mediated proteolysis genes such as *CUL1* to regulate growth and development.

A recent study has demonstrated that ubiquitin-specific proteases *UBP12* and *UBP13* are involved in circadian clock and photoperiodic floral regulation in *Arabidopsis* [53]. Double mutants of *ubp12* and *ubp13* display early flowering and short periodicity of circadian rhythms. In our study, the relative expression trend of *UBC12* was consistent with that of *COP1*.

In general, ubiquitin-mediated proteolysis pathway (PATH:ko04120)-related genes displayed much higher expression levels at YSS than at other stages, with the lowest expression observed at FBS. This trend suggests that elevated expression of these ubiquitin-mediated proteolysis pathway-related genes at YSS promotes the YSS to ASS change, and that lowered expression at FBS triggers the transition from vegetative to reproductive stages.

Many plant physiological processes are regulated by photoperiod signals [54]. Various gene expression changes reportedly induce formation of flower buds from apical buds through photoperiod induction. *GLP1*, a leaf protein-encoding gene [55], shows circadian oscillations in short-day plant *Pharbitis nil* [56] and long-day plant *Sinapis alba* [57], with the gene detected specifically in cotyledons and leaves. In maize, transcripts of *ZmGLP1* were found to be abundant in young leaves, but less frequent in other tissues and organs, such as mature leaves, young tassels, immature kernels, and stalks [58]. In our study, the *GLP1* gene was expressed at high levels during YSS and ASS; it was expressed at low levels during FBS and FS, however, being barely detectable at FS.

Relative expression of *AP1* and *AGL8* genes, which are directly associated with flowering, was high at FBS and FS, consistent with other research reports [59], [60]. The combined results of our qRT-PCR analysis confirm the reliability of the transcriptome sequencing results.

Temperature stress can delay or advance flowering. For example, growth temperatures above a finely tuned threshold can rapidly trigger flowering. *HSP70* plays an important regulatory role in the transition from vegetative to reproductive growth for photoperiodic control of flowering [61]. In our study, the relative expression of *HSP70* was reduced during *Am. tricolor* growth and flowering.

## Discussion

### Feasibility of Illumina paired-end sequencing and assembly for non-model species with unsequenced genomes such as *Amaranthus*

Understanding the dynamics of plant transcriptomes is helpful for studying the complexity of transcriptional regulation and its impact on phenotype [19]. In recent years, next-generation sequencing technologies have enabled less costly and faster generation of large-scale sequence data compared with conventional Sanger sequencing. High-throughput mRNA sequencing technology is especially suitable for gene expression profiling in non-model organisms that lack genomic sequence data [62]. Sequencing lengths reach 2×100 bp with an Illumina HiSeq 2000 paired-end 100-bp (PE 100) system, which is suitable for analysis of 200–5,000-bp lengths. This system is the most efficient and accurate approach to demarcate the boundaries of transcription units of genes and complements other methods for transcriptome studies [63]. We obtained transcriptomic data from the developmental stages YSS, ASS, FBS, and FS of *Am. tricolor* in the most systematic, thorough manner possible using the Illumina HiSeq 2000 PE 100-bp sequencing platform. At least 6 Gbp of high-quality clean reads were obtained per sample. After further gap filling and exclusion of unigenes less than 200-bp long, 99,312 unigenes containing no unknown bases (“N”) were obtained. The number of generated unigenes was more than that reported for plants such as *Ipomoea batatas* [62], *Dimocarpus longan* [64], *Phyllostachys heterocycla* [65], *Sesamum indicum* [66], *Taxus chinensis* [67], *Camellia sinensis* [68], and *Gossypium hirsutum* [69] using the same technology. Compared with previous studies, generated sequences produced shorter unigenes (mean  = 831.65 bp) than those assembled from *T. chinensis* (1,077 bp) [67], *Poncirus trifoliata* (1,000 bp; from MPSS), and *Jatropha curcas* (916 bp; from 454) [70], but they were longer than those generated from *I. batatas* (581 bp) [62], *Phyllostachys heterocycla* (612 bp) [65], *S. indicum* (629 bp) [66], *C. sinensis* [68], *Fagopyrum* (341 bp; 454) [71], and maize (218 bp; 454) [72]. More importantly, 26,637 (26.86%) of the assembled unigenes were longer than 1,000 bp. These results demonstrate that the sequencing data was of sufficient quantity and quality to ensure the accuracy of the sequence assembly. They also demonstrate that Illumina HiSeq 2000 PE100 sequencing technology can be an effective tool for gene discovery in non-model organisms [62].

Among the 99,312 generated unigenes, 43,088 (about 43.39%) were successfully annotated, suggesting their relatively conserved functions. Most unigenes were of unknown function, however, suggesting that *in vitro* flowering in *Am. tricolor* is more complex than in *Arabidopsis*. This relative sparsity of identifiable genes may also be due to the lack of a complete genomic or transcriptomic sequence set for *Am. tricolor* to use as a reference, increasing the difficulty of unigene annotation in this study. Our data provide important new insights into flowering in *Am. tricolor* and should facilitate further studies of *Am. tricolor* genes and their functions.

### Regulatory role of photoperiod during *Am. tricolor in vitro* plantlet growth and flowering

The developmental transition from nutritional growth to reproductive growth phrases is controlled by various floral integrators [55], [56], [73], [74]. The photoperiod-dependent flowering pathway is the main flowering pathway, with interactions between light and an internal timekeeping mechanism—the circadian clock—regulating photoperiodic flowering [44], [45]. Because they cannot respond to day length, plant photoperiod mutants are unable to flower normally in nature [75], [76]. Long days can accelerate floral transition in *Arabidopsis*. In the model plant *Arabidopsis*, circadian clock-related genes have been found to regulate the floral transition. Constitutive overexpression of *CCA1* delays flowering under short-day conditions [46], [47], whereas *elf3-1*, *cca1* and *lhy* mutations accelerate flowering [44], [48]. In addition, *XTH9* is expressed at different development stages of *Arabidopsis*, including differentiation of vegetative and reproductive meristems and cell elongation of inflorescence stems [51]. In our study, 196 unigenes involved in the plant circadian rhythm (PATH:ko04712) pathway were obtained from the transcriptome data, including genes such as *XTH9*, *XTH33*, *ELF3*, *LHY*, *PHYA*, *PHYB*, *PIF3*, *CRY1*, and *CRY2*. The results of our analysis indicate that photoperiod plays an important regulatory role during *Am. tricolor in vitro* plantlet growth and flowering. The qRT-PCR expression analysis confirmed the participation of these related genes in this process.

In general, ubiquitin-mediated proteolysis pathway (PATH:ko04120)-related genes showed much higher gene expressions at YSS than other stages, with the lowest expression levels observed at FBS. This result suggests that elevated expression of these genes at YSS promotes the YSS to ASS change, while reduced expression at FBS triggers the transition from vegetative to reproductive stages.

### Importance of the ubiquitin-mediated proteolysis pathway during *Am. tricolor in vitro* plantlet growth and flowering

Plant flowering is regulated by multiple external and internal signals, including photoperiod, temperature, hormone, and age-related signals. These signals ultimately converge upon the floral pathway integrators, a group of genes that are turned on or off to determine flowering time [77]. Numerous studies suggest that ubiquitin-mediated proteolysis plays an important role in flowering time regulation in response to photoperiod. Based on studies examining ubiquitination-mediated protein control of photoperiodic flowering [50], [76], the photomorphogenic repressors *COP1* and *DET1* are involved in light-controlled gene expression and developmental programs, while ubiquitination controls photoperiodic flowering [78]. In rice (*Oryza sativa*), the *COP1* ortholog *PPS* has been confirmed to promote the change from juvenile to adult phases and to suppress the transition from vegetative to reproductive stages [52], while the *pps-1* mutant is able to prolong the juvenile phase and to promote early flowering. In our study, changes in relative expression of *COP1* imply that high expression at YSS promotes the change from YSS to ASS and that reduced expression at FBS stimulates the transition from vegetative to reproductive stages. A recent study has demonstrated that ubiquitin-specific proteases *UBP12* and *UBP13* interact to regulate circadian clock and photoperiodic flowering in *Arabidopsis* [53]. In our study, 259 unigenes involved in the ubiquitin-mediated proteolysis pathway (PATH:ko04120) were obtained from the transcriptome data, suggesting the importance of this pathway during *Am. tricolor in vitro* flowering. According to the qRT-PCR analysis, these related genes were expressed more strongly at YSS than at other stages, with the lowest expression observed during FBS. This result indicates that high expression of ubiquitin-mediated proteolysis pathway (PATH:ko04120)-related genes at YSS promotes the YSS to ASS change, while their reduced expression at FBS triggers the transition from vegetative to reproductive stages. As protein-targeted degradation factors, plant ubiquitin ligase complexes thus appear to play an important role in flowering time control.

### Role of *GLP1* during *Am. tricolor in vitro* plantlet growth and flowering

Many plant physiological processes are regulated by photoperiod signals [54]. Various gene expression changes reportedly induce formation of flower buds from apical buds through photoperiod induction. *GLP1*, a leaf protein-encoding gene [55], shows circadian oscillations in short-day plant *Pharbitis nil* [56] and long-day plant *Sinapis alba* [57], with the gene detected specifically in cotyledons and leaves. In maize, transcripts of *Zm*GLP1 were found to be abundant in young leaves, but less frequent in other tissues and organs, such as mature leaves, young tassels, immature kernels, and stalks [58]. In our study, the *GLP1* gene was expressed at high levels during YSS and ASS; it was expressed at low levels during FBS and FS, however, being barely detectable at FS. This result suggests that *GLP1* plays an important regulatory role during *Am. tricolor in vitro* plantlet growth and flowering.

### Role of *HSP70* during *Am. tricolor in vitro* plantlet growth and flowering

Temperature stress can delay or advance flowering. For example, growth temperatures above a finely tuned threshold can rapidly trigger flowering. *Hsp70* plays an important regulatory role in the transition from vegetative to reproductive growth for photoperiodic control of flowering. In transgenic *Arabidopsis* plants, overexpression of *Heat Shock-induced Gene 1* (*HSG1*), a grape *Bcl-2-associated athanogene* (*BAG*), promotes floral transition by activating *CO* in the photoperiod pathway [79]. In contrast, other flowering-related proteins regulating gibberellin, autonomous, and vernalization pathways are not altered by *HSG1* overexpression. The BAG protein has a conserved BAG domain that interacts with the ATPase domain of Hsp70/HSC70, inducing early floral transition [80]. HSG1 proteins promote meristem transition from vegetative to reproductive growth, resulting in early flowering. In contrast to previous studies, *HSP70* expression was reduced in our transcriptome sequencing- and qRT-PCR-based study. We propose two possible reasons for the conflicting results. First, the plantlets were cultured in different environments. Our plantlets were grown at a constant temperature (25±1°C); in contrast, cultivation in previous studies involved 12-day-old *Arabidopsis* plants grown at 12°C and then shifted to 27°C [61] or *Vitis vinifera* samples heat-treated at 45°C for 60 min [81]. The thermal stimulation in those studies may have induced the significant gene expression level increase. Second, we detected an HSP70 protein encoded by a different *HSP70* family member than those of previous studies. Further research on the role of *HSP70* during *Am. tricolor in vitro* plantlet growth and flowering is consequently needed.

## Conclusions

In this study, we generated the first large-scale dataset of the *Am. tricolor* transcriptome. Functions, classifications, and metabolic pathways of genes expressed in *Am. tricolor* were revealed for the first time. A total of 35, 17,184, 274, and 206 genes were specifically expressed in YSS, ASS, FBS, and FS, respectively, with qRT-PCR analysis of related genes indicating their possible roles in *Am. tricolor in vitro* plantlet growth and flowering. Analysis of the generated transcriptome dataset provides new insights into molecular mechanisms of *in vitro* flowering in *Am. tricolor*.

## Materials and Methods

### Plant materials for transcriptome sequencing

**Figure 10:**
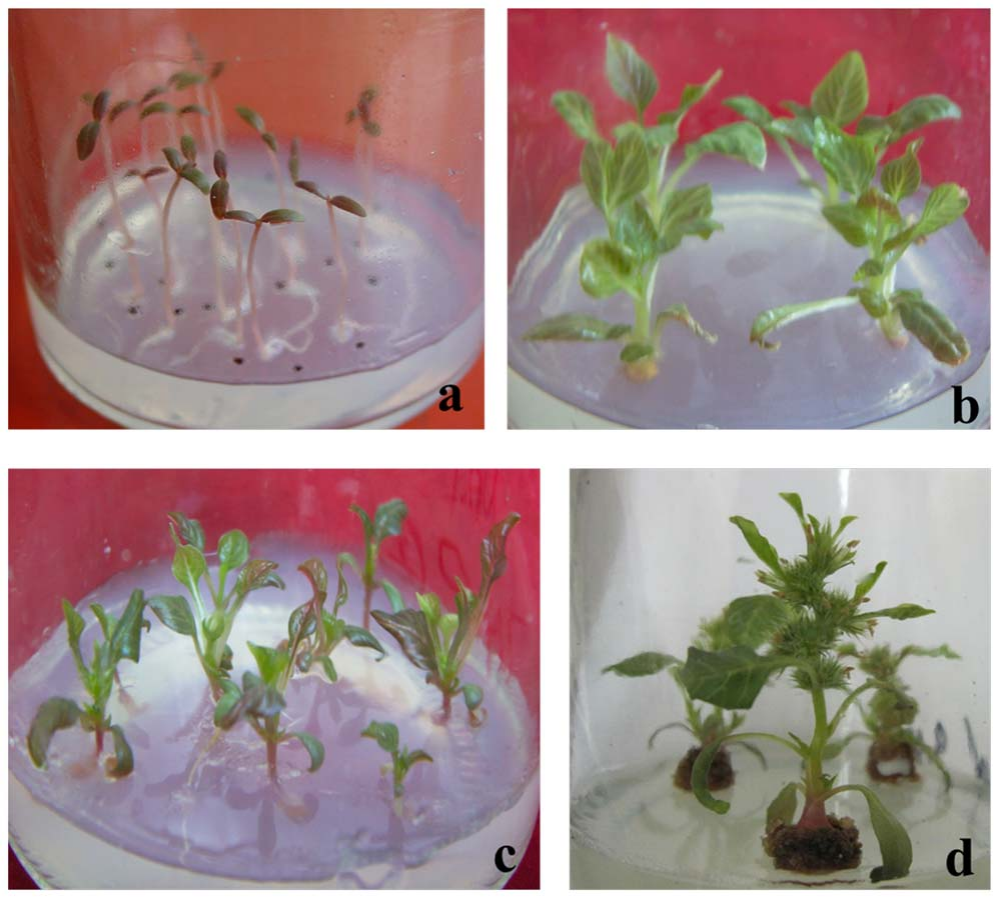
*In vitro* plantlets of *Am. tricolor* from four developmental stages, (a) YSS. (b) ASS, (c) FBS, (d) FS.

*In vitro* plantlets of *Am. tricolor* from four developmental stages—young seedling stage (YSS), adult seedling stage (ASS), flower bud stage (FBS), and flowering stage (FS)—were cultured using previously published methods [13] (Figure 10). The young seedling stage is represented by 7-day-old plantlets germinated from *in vitro* seeds. Two rounds of propagation leads to the adult seedling stage. During the flower bud stage, small flower buds have formed, while the flowering stage corresponds to the point at which *in vitro* plants are in full bloom.

### Total RNA extraction and quality detection

Total RNAs were extracted from each sample using Trizol reagent (Invitrogen, USA) and treated with DNase I to remove genomic DNA. Extracted RNAs were quantified using a NanoDrop 2000 spectrophotometer (Thermo, USA) and checked for integrity on an Agilent 2100 Bioanalyzer (Agilent Technologies) by denaturing agarose gel electrophoresis with ethidium bromide staining. Only RNA samples with A_260_/A_280_ ratios between 1.9 and 2.1, RNA 28S/18S ratios higher than 1.0, and RNA integrity numbers ≥8.5 were used in subsequent analyses; at least 15 µg of total RNA (≥300 ng µl^−1^) was used per analysis.

### cDNA library construction, quality detection, and Illumina sequencing

The cDNA library of each sample was constructed according to the instructions given in “Preparing Samples for Sequencing of mRNA Test Kits” (Illumina). Products were checked to verify the cDNA library quality and fragment length using an Agilent 2100 DNA 1000 Kit. Sequencing of the resulting cDNA library was carried out on an Illumina HiSeq 2000 paired-end 100-bp (PE 100) system.

### Data filtering and *de novo* assembly

Following sequencing of each cDNA library, the raw sequencing data were transformed by base calling into sequence data termed raw data or raw reads. Adaptor fragments, duplicated sequences, low-quality reads with ambiguous bases (“N”), and reads with more than 10% of Q-values <20 bases were removed from the raw reads to yield the clean reads required for analysis. *De novo* transcriptome assembly of these short reads was performed using the Trinity assembly program, which first combined reads with a certain length of overlap to form longer fragments, called contigs, having no ambiguous bases. The reads were mapped back to the contigs, and paired-end reads were used to detect contigs arising from the same transcript as well as the distances between these contigs. Next, Trinity connected the contigs, using ‘N’ to represent unknown sequences, to yield scaffolds. Paired-end reads were then reused to fill in the gaps between the scaffolds, yielding sequences that had the fewest Ns and that could not be extended on either end. These resulting sequences were designated as unigenes. The raw data were deposited in the NCBI SRA under accession numbers SRR924089, SRR924090, SRR924091, and SRR924092.

### Gene annotation, classification, and metabolic pathway analysis

ORFs of unigene sequences greater than 200-bp long were identified using getorf software (http://emboss.bioinformatics.nl/cgi-bin/emboss/getorf). Using BLASTX, the unigene sequences were then searched (*E*-value <1×10^−5^) against Nr, Swiss-Prot, KEGG, and COG databases. The best results from the alignment were used to predict unigene coding regions and direction (i.e., 5' to 3'). When results from different databases conflicted, annotations were applied according to the following order: Nr > SwissProt > KEGG > COG. Unigene annotations were used to provide information on expression patterns and functions of the identified genes.

The gene2go (ftp://ftp.ncbi.nlm.nih.gov/gene/DATA/) program was used in conjunction with the Nr annotations to obtain unigene GO annotations, with Excel 2010 (Microsoft Corporation) then used for all GO functional classifications.

Collections of Clusters of Orthologous Genes (COGs) provide indispensable tools for comparative genomic analysis, evolutionary reconstruction and functional annotation of new genomes, and various genome-wide evolutionary analyses [70]. To evaluate the completeness of our transcriptome library and the effectiveness of our annotation process, the unigene sequences were searched for the possible functions involved in COG classifications.

A metabolic pathway analysis was performed using the KEGG database and related software applications (http://www.genome.jp/kegg/kegg4.html), which form a bioinformatics resource for linking genomes to living organisms. KEGG pathway maps, BRITE functional hierarchies, and KEGG modules are represented in a generic way to be applicable to all organisms. The KEGG Orthology (KO) system is the basis for this representation. Once unigenes are assigned KO identifiers, or K numbers, organism-specific pathway maps and BRITE functional hierarchies are automatically generated. Within the KEGG databases, the PATHWAY database contains information on networks of molecular interactions within cells, as well as variations specific to particular organisms. Consequently, the acquired *Am. tricolor* unigenes based on the KEGG database were further extended by assigning them to metabolic pathways using BLASTX.

### Differential gene expression analysis

After RPKM-normalization of unigene expression levels [20], [41], analysis of differentially expressed genes was carried out between FBS and YSS, FBS and ASS, and FBS and FS using DESeq software [43], with a *p*-value cut-off of <0.05 applied after adjustment for multiple testing based on the Benjamini and Hochberg false discovery rate method.

### qRT-PCR analysis

qRT-PCR analysis based on gene-specific primers that were designed with DNAMAN 6.0 was used to verify candidate unigenes having potential roles during *Am. tricolor in vitro* plantlet growth and flowering. RNA samples used for the qRT-PCR analysis were the same as those used for the cDNA library construction. Purified RNA was reverse-transcribed to cDNA using a SYBR PrimeScript RT-PCR kit (Perfect Real Time; Takara, China). qRT-PCR reactions were performed in triplicate with three biological replicates per sample according to the SYBR Green I Master Mix protocol (Takara) on a LightCycler 480 qPCR instrument (Roche, Switzerland). NormFinder (http://moma.dk/normfinder-software) was used to analyze the stability of candidate unigene expression. The *HERC* gene was used as an internal reference gene for calculation of relative expressions of the candidate unigenes.

Gene names, primer sequences, product sizes, PCR efficiencies, and annealing temperatures are given in File S2.

## Supporting Information

File S1 **KEGG classifications of genes expressed during** ***Am. tricolor in vitro*** **plantlet growth and flowering.**
(XLS).

File S2 **qRT-PCR primers used to analyze candidate unigenes expressed during** ***Am. tricolor in vitro*** **plantlet growth and flowering.**
(XLS)
